# Serum microRNA Levels in Diabetes Mellitus

**DOI:** 10.3390/diagnostics11020284

**Published:** 2021-02-11

**Authors:** Rodolfo Mastropasqua, Rossella D’Aloisio, Erica Costantini, Annamaria Porreca, Giada Ferro, Daniele Libertini, Marcella Reale, Marta Di Nicola, Pasquale Viggiano, Gennaro Falconio, Lisa Toto

**Affiliations:** 1 Institute of Ophthalmology, University of Modena and Reggio Emilia, 41121 Modena, Italy; rodolfo.mastropasqua@gmail.com; 2 Ophthalmology Clinic, Department of Medicine and Science of Ageing, University “G. d’Annunzio” Chieti-Pescara, Via dei Vestini 31, 66100 Chieti, Italy; giadaferro90@gmail.com (G.F.); daniele.libertini@hotmail.com (D.L.); pasquale.viggiano90@gmail.com (P.V.); gennarofalconio@libero.it (G.F.); l.toto@unich.it (L.T.); 3 Department of Medical, Oral and Biotechnological Science, University “G. d’Annunzio” Chieti-Pescara, Via dei Vestini 31, 66100 Chieti, Italy; erica.costantini@unich.it (E.C.); marcella.reale@unich.it (M.R.); mdinicola@unich.it (M.D.N.); 4 Department of Economic Studies, University “G. d’Annunzio” Chieti-Pescara, Viale Pindaro, 65100 Pescara, Italy; annamaria.porreca@unich.it

**Keywords:** diabetes mellitus, diabetic retinopathy, serum microRNA

## Abstract

The aim of our study is to evaluate the serum circulating levels of some miRNA, such as hsa-let-7b-5p, hsa-let-7a-5p, hsa-miR-320b, hsa-miR-23a-3p, hsa-miR-27a-3p, hsa-miR-15a-5p, and hsa-miR-495-3, in diabetic patients without diabetic retinopathy (DR), diabetic patients with DR, and, healthy subjects in order to find reliable and reproducible biomarkers for DR. A total of 45 subjects underwent serum sampling for miRNAs evaluation and a complete ophthalmologic examination, including microperimetry and widefield swept source optical coherence tomography angiography (OCTA). Total circulating RNA was isolated from patients using the miRNeasy Serum/Plasma Kit. Serum miRNA expression levels were significantly different in the three groups. In detail, circulating hsa-miR-15a-5p levels were significantly reduced in both diabetic patients without DR and diabetic patients with DR (*p* = 0.027). Serum hsa-miR-495-3p was lower in diabetic patients with DR and diabetic patients without DR (*p* = 0.049). Hsa-miR-23a-3p serum expression levels were significantly lower in diabetic patients with DR and diabetic patients without DR (*p* = 0.013). Significant associations of miRNAs with anatomical/perfusion parameters and functional parameters were observed in the diabetic groups. We find evidence of damage in progression biomarkers in DR that are evidently early in patients with diabetes without DR. Serum miRNAs levels are considered to have strong potential as a novel biomarker for the early detection of DR in subjects suffering from diabetes and could represent noninvasive target therapies to block the progression of the disease at the early stages.

## 1. Introduction

Diabetic retinopathy (DR) is the most frequent complication of diabetes mellitus (DM), characterized by microvascular changes in the retina [1]. Due to the increased incidence of diabetes as a global epidemic disease, DR is estimated to occur for a third of people with diabetes, becoming the leading cause of preventable blindness in the working-age population in developed countries [2]. In the USA, it has been estimated that 40% of people with type 2 diabetes mellitus (T2DM) and 86% with type 1 diabetes (T1DM) will develop DR [3,4].

Diabetic retinopathy is divided into two major forms: nonproliferative (NPDR) and proliferative (PDR), named for the absence or presence of abnormal new blood vessels emanating from the retina. Treatment options are lacking in early-stage nonproliferative retinopathy [1].

Currently, with advances in retinal imaging, optical coherence tomography angiography (OCTA) is a useful tool to investigate in detail the morphological and perfusion changes of retinal and choroidal vasculature in DR patients [5]. Additional information about the precise topographical distribution of such DR-related changes, like vessel density (VD), intercapillary spacing, or the vessel diameter index, could help the characterization of the disease [6].

Despite the advances in multimodal retinal imaging, further research is required for the understanding of the pathogenesis, outcome, and treatment of diabetic retinopathy.

Interestingly, several studies have suggested the involvement of microRNAs (miRNAs) in DR [7,8]. miRNAs are single-stranded (18–23 bp), noncoding RNAs that have been described to be involved in several biological processes that regulate gene expression at the post-transcriptional level [9]. Previous studies have reported an up- or downregulation of some circulating miRNAs in patients affected by DM complicated with DR when compared with healthy subjects, suggesting an important role as predictive biomarkers for progression to advanced and proliferative forms [7,8]. The link between miRNAs and DR is represented by the important regulatory role of glucose homeostasis, such as peripheral insulin signaling, β cells function, and phenotype maintenance [10,11].

The aim of our study is to evaluate the serum circulating levels of some miRNAs, such as hsa-let-7b-5p, hsa-let-7a-5p, hsa-miR-320b, hsa-miR-23a-3p, hsa-miR-27a-3p, hsa-miR-15a-5p, and hsa-miR-495-3, in diabetic patients without DR, diabetic patients with DR, and healthy subjects in order to find reliable and reproducible biomarkers for DR. Moreover, the secondary aim is to correlate differentially expressed miRNAs to anatomical/functional and perfusion parameters.

## 2. Materials and Methods

### 2.1. Study Participants

In this observational cross-sectional study, 45 subjects, aged between 45 and 65 years, who were enrolled at the Ophthalmology Clinic, University “G. d’Annunzio”, Chieti-Pescara, Italy, were divided into three groups. In the first group, patients with T2DM and no sign of DR were included (DM without DR); in the second group, patients with T2DM and signs of nonproliferative DR (NPDR; including the early-moderate stage of NPDR) were included (DM with NPRD), and the third group, with 15 healthy subjects, was considered the control. Power analysis demonstrated that 15 patients in each group give a power of 80% to detect a mean difference of 30% in the miRNA levels between the DM without DR group and the healthy group, at an alpha level of 0.05. The study was approved by the Institutional Review Board (IRB; Department of Medicine and Science of Ageing, University “G. d’Annunzio” Chieti-Pescara) and adhered to the tenets of the Declaration of Helsinki. IRB-approved informed consent was obtained from all subjects. The exclusion criteria of both T2DM and healthy subjects were (1) any history of ocular surgery (included intravitreal injections); (2) laser treatment; (3) history of any ocular disease. All subjects, recruited between February 2019 to January 2020, underwent a complete ophthalmologic examination, including the measurement of best-corrected visual acuity (BCVA), intraocular pressure (IOP), and dilated ophthalmoscopy. Moreover, all patients underwent microperimetry (MP) by means of an MP-3 microperimeter (Nidek Technologies, Padova, Italy), widefield swept source OCTA using a PLEX Elite 9000 device (Carl Zeiss Meditec Inc., Dublin, CA, USA), and serum sampling for miRNAs evaluation.

### 2.2. Microperimetry

**Figure 1:**
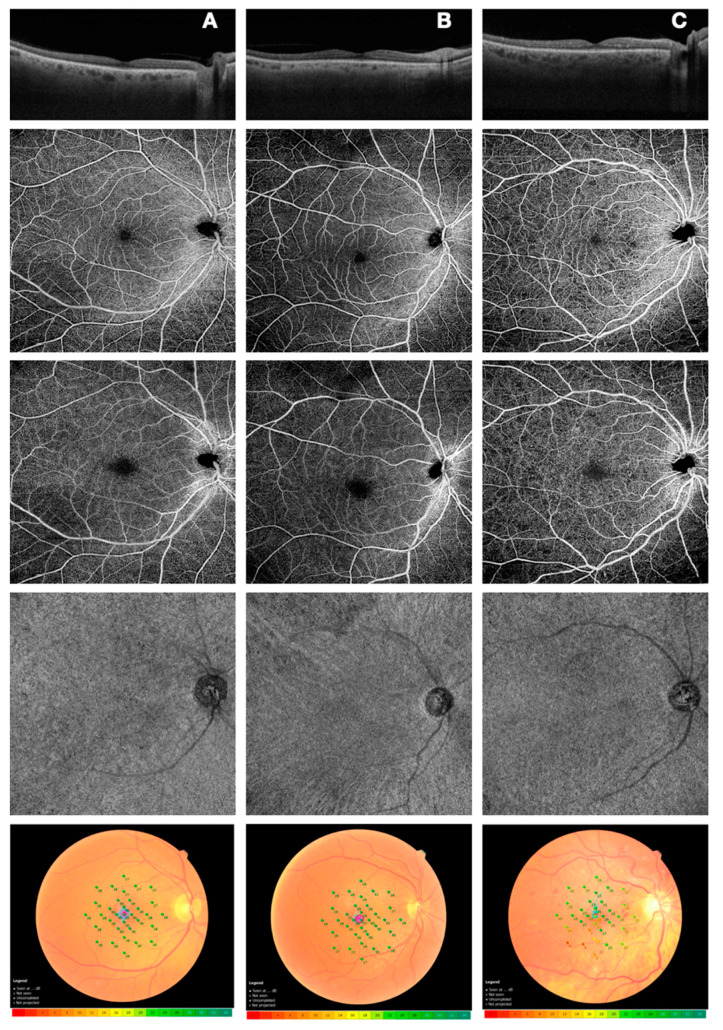
Comparison of OCT (first line), widefield OCTA (SCP, second line; DCP, third line; CC, third line), and microperimetry (fourth line) among the three groups (healthy (**A**); DM without DR (**B**); DM with NPDR, (**C**)). Abbreviations: OCT, optical coherence tomography; OCTA, optical coherence tomography angiography; SCP, superficial capillary plexus; DCP, deep capillary plexus; CC, choriocapillaries; DM, diabetes mellitus; DR, diabetic retinopathy; NPDR, nonproliferative diabetic retinopathy.

To perform microperimetry, all patients were dilated with tropicamide 1% eye drops. This test is routinely carried out with an automated eye-tracking system. To assess central macular retinal sensitivity, differential light threshold values were compared by calculating the mean of the 4°, 8°, and 20° of the macular area, which was averaged automatically by the MP-3 software program for the mean sensitivity in a polygon (Figure 1).

### 2.3. Optical Coherence Tomography Angiography

Patients underwent OCTA imaging using the widefield PLEX Elite 9000 device (Carl Zeiss Meditec Inc., Dublin, CA, USA), which uses a swept laser source with a central wavelength of 1050 nm (1000–1100 nm full bandwidth) and operates at 100,000 A-scans per second. For each eye, a 12 × 12 mm OCTA volume scan was acquired. FastTrack motion correction software was used while the images were acquired. Poor quality images (signal strength index (SSI) < 8), with either significant motion artifacts or extensive incorrect segmentation, were excluded and repeated. For all the participants, one or both eyes were imaged separately three times each, and the best quality image from each eye was selected to be analyzed in the study. All selected images were carefully visualized by two retinal specialists, in consensus, to ascertain the correctness of segmentation and, in the case of erroneous recognition by the software of the position of the boundaries of the inner limiting membrane (ILM) and retinal pigment epithelium (RPE), manual correction was performed using the segmentation and propagation editing tool from the device. Subsequently, automatic segmentation by the PLEX Elite 9000 device was used to define vascular beds, obtaining three depth-resolved retinal slabs: the superficial capillary plexus extends from the internal limiting membrane (ILM) to the inner plexiform layer (IPL), the deep capillary plexus extends from the IPL to the outer plexiform layer (OPL), and the choriocapillaris (consisting of a 20-μm thick uniform layer) extends 29 μm below the RPE to 49 μm below the RPE. Vessel density (VD) of the superficial capillary plexus (SCP), deep capillary plexus (DCP), and choriocapillaris (CC) was calculated (Figure 1). The details of the methods used to quantify these variables have been previously described [12]. For each eye, OCTA scans, segmented at the SCP, DCP, and CC levels, were imported into ImageJ software version 1.50 (National Institutes of Health, Bethesda, MD, USA; available at http://rsb.info.nih.gov/ij/index.html). Each SCP and DCP en face image was binarized with the default binarization method to calculate perfusion density, as previously shown. To evaluate CC and VD, en face images were binarized using the Phansalkar method, and then, the obtained images were processed with the “analyze particles” command, as previously described [5]. The DCP and CC directly beneath major superficial retinal vessels were excluded from the analysis to eliminate potentially confounding shadows or projection artifacts. Successively, the SCP, DCP, and CC images obtained after binarization were analyzed. Quantitative analysis was performed in two different regions: (i) the macular region, which was defined as three concentrical circular annuli around the fovea, with diameters of 1.5, 3, and 5 mm (respectively called “foveal area”, “parafoveal area”, and “perifoveal area”); (ii) the periphery region, which was assessed in three tangential circles (“superior ring”, “temporal ring”, and “inferior ring”) to the parafoveal area, with diameters of 3 mm.

### 2.4. Serum miRNAs Sampling and Real-Time Quantitative Polymerase Chain Reaction (RT-qPCR) Analysis

Total circulating RNA (tRNA) was isolated from patients’ serum using the miRNeasy Serum/Plasma Kit (QIAGEN, Hilden, Germany), according to the manufacturer’s instructions. Quality and quantity of microRNAs were assessed with NanoDrop™ 2000/2000c (Thermo Scientific, Waltham, MA, USA). Equal amounts (5 ng) of miRNA were used for reverse transcription (RT) using the miRCURY LNA RT Kit (QIAGEN, Hilden, Germany) and, for amplification by qPCR, using the miRCURY LNA SYBR^®^ Green PCR Kit (QIAGEN, Hilden, Germany) with CFX Real-Time PCR Detection Systems (Bio-Rad, Hercules, CA, USA). Briefly, hsa-let-7a-5p, hsa-let-7b-5p, hsa-miR-23a-3p, hsa-miR-27a-3p, hsa-miR-15a-5p, hsa-miR-320b, and hsa-miR-495-3p miRNAs were evaluated in duplicate for each sample and compared with hsa-miR-423-3p, identified as the most stably expressed and used as a reference. The miRNAs were selected from miRCURY LNA miRNAs PCR assays (QIAGEN, Hilden, Germany), and the miRBase accession numbers are reported in Table 1. The expression was calculated according to the 2^−ΔΔCt^ method.

### 2.5. Statistical analysis

We calculated that 15 patients per group gives a power of 80% to detect a mean difference of 30% in the miRNA-15 between the DM without DR group and the healthy group, at an alpha level of 0.05. Descriptive statistics: median and 1st and 3rd quartiles were calculated to characterize the study variables. Normal distribution was verified with the Shapiro–Wilk test, and, for all variables, the null hypothesis of normality was not verified, with a significance level of 95%. The Kruskal–Wallis test was applied to investigate whether there were significant differences between healthy, DM without DR, and DM with NPRD groups of patients. When the nonparametric analysis of variance resulted in a significant difference between groups, Dunn’s posthoc test, with a Bonferroni correction test, was used to compute multiple pairwise comparisons. Radar chart analyses were used to visualize the expression patterns of the miRNAs. A radar chart is a graphical method of displaying multivariate data in the form of a two-dimensional chart of three or more quantitative variables represented on axes starting from the same point. Spearman’s rho correlation coefficient, with listwise-deletion, was calculated to compare the relationship of the continuous parameters. R statistical environment version 3.6 was used for all data analysis.

## 3. Results

### 3.1. Anatomical/Perfusion and Functional Parameters

A total of 45 subjects were enrolled in the study. The demographic characteristics, in terms of gender and age, were not different between the three groups of patients: the healthy group (mean age of 60.00; 33.33% males, 66.67% females); the DM without DR group (mean age of 68.50; 33.33% males; 66.67% females); the DM with NPRD group (mean age of 61.00; 60.00% males; 40.00% females) (*p* > 0.05).

A significant difference was found among the three groups in terms of BCVA and serum glycosylated hemoglobin, as shown in Table 2.

Significantly reduced retinal sensitivity was found in the NPDR group and in the no-DR group compared with the control group in all the investigated areas (4°, 8° and 20° of the macular area; *p* = 0.002), as reported in Table 2.

Regarding widefield OCTA analysis, a significant reduction was found in both diabetic subject groups (without DR and with NPDR) in terms of vessel density at the DCP and CC level.

In detail, the lowest value of VD was detected in the inferior region of retinal midperiphery in DCP and in the foveal region in CC (*p* = 0.033; *p* = 0.001). No significant differences were assessed in SCP among the three groups.

Quantitative analysis of ophthalmologic examination, microperimetry, and OCTA is expressed as median values. For all three groups, the median (1st quartile; 3rd quartile) and *p*-value, relative between group comparisons, are shown. Statistically significant posthoc comparisons (*p* < 0.05) are reported as superscript brackets; indeed, the star (*) indicates that all groups are different from each other.

### 3.2. MicroRNA Expression Levels

Serum miRNAs expression levels were significantly different between the three groups, as reported in Table 3. In detail, circulating hsa-miR-15a-5p levels were significantly reduced in both DM without DR and DM with NPRD groups when compared with the healthy group (*p* = 0.027). Serum hsa-miR-495-3p was lower in the DM with NPDR group and the DM without DR group compared with the healthy group (*p* = 0.049). Moreover, hsa-miR-23a-3p serum expression levels were significantly lower in the DM with NPDR group and the DM without DR group in comparison with the healthy group (*p* = 0.013).

For all three groups, the median (1st quartile; 3rd quartile) and *p*-value, relative to group comparisons, are shown. Statistically significant posthoc comparisons (*p* < 0.05) are reported as superscript brackets.

**Figure 2:**
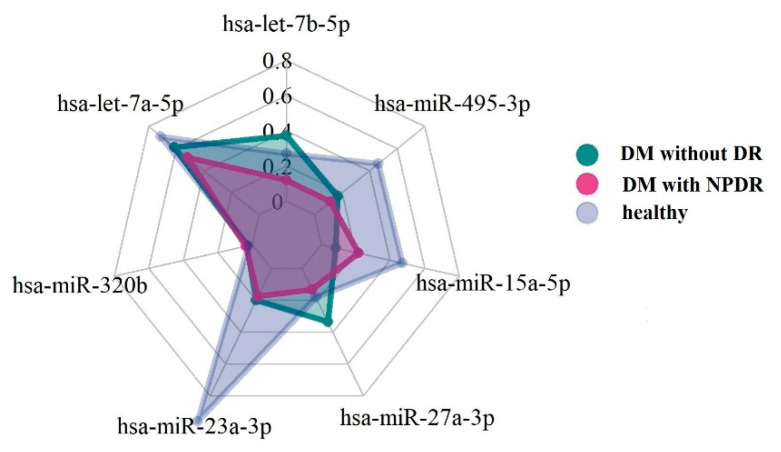
Radar plot for median values of miRNAs.

In order to visualize the expression pattern differences of the serum levels of miRNAs in the experimental groups, a radar chart representation was done (Figure 2). The graph shows how the DM without DR group has, as a median, except for hsa-miR-15a-5p, higher values of miRNAs.

### 3.3. Correlation analysis

The pairwise correlation matrix between items was computed (based on the Spearman correlation method) to evaluate the association of circulating miRNAs with anatomical/perfusion and functional parameters in our diabetic groups. Only statistically significant correlations are reported in Table 4.

## 4. Discussion

In this observational study, we quantified the blood levels of some miRNAs in patients suffering from DM, with and without signs of DR, and we correlated them with functional and anatomical/perfusion parameters.

Commonly, miRNAs are expressed in all human cell-types and are involved in cell growth, differentiation, and apoptosis [13] through the modulation of about 60% of the protein-coding genes in mammalians [14]. miRNAs molecules are responsible of a wide range of physiological and pathological processes and have been found to be stable in cell-free body fluids like serum [15].

Interestingly, different studies have shown that some miRNAs are associated with glucose homeostasis, mostly in association with genes for diabetes-relevant pathways like insulin signaling [16,17,18]. Furthermore, it has been shown that some miRNAs can regulate the synthesis and secretion of insulin and play an important role in blood glucose balance and in the pathophysiology of DR-related micro- and macrovascular complications [14,18,19].

In our study, levels of different miRNAs, such as hsa-let-7b-5p, hsa-let-7a-5p, hsa-miR-320b, hsa-miR-23a-3p, hsa-miR-27a-3p, hsa-miR-15a-5p, and hsa-miR-495-3p, were detected using serum samples from patients suffering from DM, with and without DR, and healthy controls. Moreover, hsa-miR-15a-5p has been identified as a protective factor for its anti-inflammatory and antiproliferative roles [20]. Furthermore, the miR15 family seems to have a role in protein mechanisms involved in insulin signaling, causing an alteration of insulin secretion [21]. Our findings, in accordance to the literature, showed statistically significant differences in hsa-miR-15a-5p levels in patients with DM without DR and with NPDR compared to healthy controls, thus suggesting its potential predictive role in diabetic patients that have yet to develop signs of DR [22,23,24,25,26]. Kamalden at al. have already described that in the early stages of diabetes, insulin production promotes the activation of hsa-miR-15a-5p expression, stimulating β cells [24]. Moreover, hsa-miR-15a-5p dysregulation promotes ROS production, causing oxidative stress and apoptotic cell death due to inhibition of the phosphoinositide 3-kinase signaling pathway, contributing to DR disease progression [24].

Additionally, hsa-miR-23a-3p, well-known to represent one of the main families of miRNAs involved in the regulation of the T-cell immune response, showed significant reduction levels in diabetic patients and even more in patients who developed DR. The hsa-miR-23a-3p expression is linked to the inhibition of the NF-κB pathway, with the consequent blockage of the proinflammatory response and cellular angiogenic potential [25,26]. Furthermore, its association with diabetic disease is due to its involvement in the regulation of fasting glucose levels and as a suppressor of tumor migration in the case of hepatocellular carcinoma [26]. A decreased level of circulating hsa-miR-23a-3p has been considered responsible for metabolic mechanism dysregulation in diabetic retinopathy [27]. Likewise, Yang et al. detected low serum levels of hsa-miR-23a-3p, identifying in it a valuable biomarker for the early diagnosis of T2DM [27].

hsa-miR-495-3p is involved in promoting the development of normal tissues and regulating the proliferation, apoptosis, and metastasis of cancer cells [28]. In our patients, a significant reduction of hsa-miR-495-3p was detected, with a decrease of 69% for subjects with DM without DR and 74% for DM with NPDR patients compared to healthy controls. The finding of our study, with hsa-miR-495-3p downregulation, strengthens its predictive role in the development of DM-related retinal damage, as demonstrated by different studies [29,30].

Indeed, at the retinal level, Zhang et al. have shown that miR495 is involved in the regulation of retinal ganglion cell (RGC) apoptosis induced by hyperglycemia. Studies have reported that downregulation of hsa-miR-495-3p has a protective role for retinal ganglion cells from hyperglycemia-induced apoptosis, while the overexpression of hsa-miR-495-3p has the opposite effect [28]. RGC death is one of the main steps in the pathogenesis and development of diabetic retinopathy [30]. Moreover, in T2DM patients, an association between downregulated hsa-miR-495-3p levels and the insulin secretion process has been found [31].

Some studies have underlined the overexpression of hsa-miR-27a-3p and let-7 family genes, both related to glucose metabolism and insulin sensitivity [30,31]. On the contrary, our result data did not show any statistically significant differences in hsa-let-7b-5p values between the three groups.

As a secondary outcome, we aimed to investigate a possible correlation between miRNAs levels and anatomical/functional and perfusion parameters of retina/choriocapillaris.

Our data showed a significant reduction in retinal sensitivity and vessel density in diabetic patients, especially in those who developed NPDR.

Vujosevic et al. described that neurodegeneration could precede the loss of photoreceptors in diabetic retinopathy, thus underlining the importance of microperimetry in integrating this functional parameter with the morphological state of the retina [32,33].

Similarly, Sharanjeet-Kaur et al. reported that a reduction in retinal sensitivity is related to a statistically significant increase in HBA1C [34]. In our study, no significant correlations were found between retinal sensitivity (4°, 8° and 20° MP) and miRNA levels.

Of note, we quantified the perfusion density of retinal and choriocapillaris vasculature using a widefield swept source OCTA imaging device, which is able to better analyze CC due to high speed and wavelength and can visualize both central and peripheral areas in a single scan [35]. In the literature, a reduction of perfusion of the superficial and deep plexus has been proven to be in parallel with DR progression [36,37,38,39]. In previous work, a significant progressive decrease in vessel density was assessed at the retinal layer and CC in patients with DR compared with controls as retinopathy progressed in both central and peripheral retina [5]. Some authors have also described a VD decrease in subjects with a diagnosis of diabetes but not of DR [40,41,42].

In our study, we did not find any statistically significant reduction of vascular perfusion in the SCP; in contrast, significantly lower values of VD were found in CC and DCP, particularly in the lower sector of midperiphery, in both groups of diabetics, with and without DR. 

Previous works have hypothesized that hypoperfusion of the choriocapillaris could cause a reduction of oxygen supply and nutrients for retinal photoreceptors [43,44,45]. The lack of metabolic supply can induce photoreceptor loss [46]. According to the literature, we found an impairment in the DCP and CC flow in both patients with DR and diabetic patients without DR, suggesting that both retinal and choroidal circulation are affected before clinical manifestation of DR [47].

Moreover, in our study, a correlation between VD values and miRNA values was reported. Although there was no statistically significant reduction of VD in the SCP, it should be noted that hsa-let-7b-5p and hsa-miR-495-3p have a strong significant positive correlation with vessel density, thus confirming the predictive role of blood miRNA levels in DM and DR pathophysiology. The main limitation of our study is the small sample. The latter is composed of patients with diabetes, complicated or not with early-moderate NPDR. Further studies are needed, with a wider sample and with severe NPDR/proliferative DR, for better comprehension of miRNA changes in DR progression.

In conclusion, in our study, we find evidence of damage progression biomarkers in diabetic retinopathy that are present early in patients with diabetes without DR. A reduction in retinal sensitivity and retinal/choriocapillaris perfusion are indicative of the functional damage that precedes the morphological damage. Serum miRNA levels are considered to have a strong potential as a novel biomarker for the early detection of diabetic retinopathy in subjects suffering from diabetes and could represent noninvasive target therapies to block the progression of the disease at the early stages.

## Figures and Tables

**Table 1:** miRNA accession numbers.

miRNA Base ID	miRBase Accession: Mature miRNA Sequence
hsa-let-7b-5p	MIMAT0000063: 5′UGAGGUAGUAGGUUGUGUGGUU
hsa-let-7a-5p	MIMAT0000062: 5′UGAGGUAGUAGGUUGUAUAGUU
hsa-miR-320b	MIMAT0005792: 5′AAAAGCUGGGUUGAGAGGGCAA
hsa-miR-23a-3p	MIMAT0000078: 5′AUCACAUUGCCAGGGAUUUCC
hsa-miR-27a-3p	MIMAT0000084: 5′UUCACAGUGGCUAAGUUCCGC
hsa-miR-15a-5p	MIMAT0000068: 5′UAGCAGCACAUAAUGGUUUGUG
hsa-miR-495-3p	MIMAT0002817: 5′AAACAAACAUGGUGCACUUCUU
hsa-miR-423-3p	MIMAT0001340: 5′AGCUCGGUCUGAGGCCCCUCAGU

**Table 2:** Anatomical/perfusion and functional parameters. All values are expressed as median (1st quartile; 3rd quartile). *p*-value results from the nonparametric test for independent samples. For analyzing the specific sample pairs for stochastic dominance, Dunn’s test was used. To show the pairwise differences, we assigned a number to each group: healthy = (1), DM without DR = (2), DM with NPRD = (3). The star (*) indicates that all groups are different from each other; then, if not, then the number of the group from which it differs is indicated.

Variable	Healthy ^(1)^	DM without DR ^(2)^	DM with NPRD ^(3)^	*p*-Value
BCVA (logMAR)	0.00 [0.00;0.00]	0.01 [0.00;0.10]	0.15 [0.10;0.27]	<0.001 *
CMT	203 [201;204]	228 [203;236]	256 [223;276]	<0.001 *
HBA1c (%)	4.7 [4.6;4.9]	7.00 [6.8;7.0]	8.4 [6.8;11.2]	<0.001 *
4° MP (db)	29.1 [28.5;29.4]	25.1 [24.8;25.8] ^(1)^	23.4 [21.4;26.3] ^(1)^	0.002
8° MP(db)	29.1 [28.9;29.7]	26.1 [25.5;28.3] ^(1)^	23.9 [22.6;26.9] ^(1)^	0.002
20° MP (db)	28.8 [28.6;29.5]	26.1 [25.3;27.8] ^(1)^	23.6 [21.9;26.8] ^(1)^	0.002
**Vessel Density**				
Inferior ring_DCP	44.5 [42.5;47.9]	41.1 [33.5;41.5]	37.6 [31.9;40.1] ^(1)(2)^	0.033
Foveal ring_CC	74.4 [72.3;75.5]	72.3 [71.1;74.0]	67.9 [66.3;71.0] ^(1)(2)^	0.001
Parafoveal ring_CC	75.5 [72.9;76.7]	73.9 [71.8;75.0]	68.9 [67.3;70.6] ^(1)(2)^	<0.001
Perifoveal ring_CC	75.7 [73.1;76.3]	74.2 [72.3;75.2]	68.5 [67.5;70.9] ^(1)(2)^	<0.001
Superior ring_CC	73.2 [71.8;75.2]	74.6 [72.8;75.9]	68.6 [66.2;69.8] ^(1)(2)^	<0.001
Inferior ring_CC	75.3 [74.9;77.1]	74.8 [73.0;75.3]	69.7 [67.3;71.2]	<0.001 *
Temporal ring_CC	75.1 [73.4;76.6]	74.7 [72.7;76.3]	72.1 [68.2;73.8] ^(1)(2)^	0.019

HBA1c, glycated hemoglobin; CMT, central macular thickness.

**Table 3:** Expression of miRNA levels in the three groups. All values are expressed as median (1st quartile; 3rd quartile). *p*-value results from the nonparametric test for independent samples. For analyzing the specific sample pairs for stochastic dominance, Dunn’s test was used. To show the pairwise differences, we assigned a number to each group: healthy = (1); DM without DR = (2); DM with NPRD = (3).

miRNA	Healthy ^(1)^	DM without DR ^(2)^	DM with NPRD ^(3)^	*p*-Value
hsa-let-7b-5p	0.34 [0.18;0.44]	0.46 [0.29;0.74]	0.15 [0.12;0.24]	0.217
hsa-let-7a-5p	0.89 [0.56;2.06]	0.86 [0.35;1.19]	0.65 [0.35;0.94]	0.625
hsa-miR-320b	0.03 [0.03;0.03]	0.04 [0.03;0.10]	0.04 [0.02;0.07]	0.519
hsa-miR-23a-3p	1.20 [0.57;1.37]	0.29 [0.13;0.33] ^(1)^	0.22 [0.10;0.30] ^(1)^	0.013
hsa-miR-27a-3p	0.22 [0.19;0.52]	0.42 [0.22;0.74]	0.16 [0.11;0.29]	0.230
hsa-miR-15a-5p	0.59 [0.46;0.68]	0.11 [0.06;0.52] ^(1)^	0.27 [0.16;0.35] ^(1)^	0.027
hsa-miR-495-3p	0.58 [0.22;0.96]	0.18 [0.08;0.22] ^(1)^	0.15 [0.07;0.21] ^(1)^	0.049

**Table 4:** Nonparametric correlation coefficients between circulating miRNAs and anatomical/functional and perfusion variables in patients with diabetes.

miRNA	Ophthalmological Variables
hsa-let-7b-5p	Foveal ring VD_SCP
	Rho = 0.888 *
hsa-miR-15a-5p	Temporal ring VD_CC
	Rho = 0.895 *
hsa-miR-320b	BCVA (logMAR)
	Rho = 0.894 *
hsa-miR-23a-3p	Inferior ring VD_CC
	Rho = −0.963 **
hsa-miR-27a-3p	Superior ring VD_CC
	Rho = −0.945 *
hsa-miR-495-3p	Superior ring VD-SCP
	Rho = 0.972 **

Correlations were obtained using the Spearman method with listwise deletion. In the table, only statistically significant coefficients are reported. Significance codes: 0.01 “**”; 0.05 “*”. Abbreviations: VD, vessel density; SCP, superficial capillary plexus; DCP, deep capillary plexus; CC, choriocapillaris.

## Data Availability

The data presented in this study are available on request from the corresponding author
